# Active Video Game Interventions Targeting Physical Activity Behaviors: Systematic Review and Meta-analysis

**DOI:** 10.2196/45243

**Published:** 2023-05-16

**Authors:** Arlen C Moller, Caio Victor Sousa, Kelly Jihyeon Lee, Dar Alon, Amy Shirong Lu

**Affiliations:** 1 Department of Psychology Lewis College of Science and Letters Illinois Institute of Technology Chicago, IL United States; 2 Department of Health and Human Sciences Seaver College of Science & Engineering Loyola Marymount University Los Angeles, CA United States; 3 College of Arts, Media & Design Bouvé College of Health Sciences Northeastern University Boston, MA United States; 4 T.H. Chan School of Public Health Harvard University Boston, MA United States

**Keywords:** active video game, exergame, games for health, digital health, physical activity, systematic review, meta-analysis, digital game, digital health intervention, health promotion

## Abstract

**Background:**

Research on digital games designed to increase physical activity (PA), also known as exergames or active video games (AVGs), has proliferated over the past 2 decades. As a result, reviews of literature in this field can become outdated, revealing the need for updated high-quality reviews that identify overarching insights. Furthermore, given the significant heterogeneity in AVG research, study inclusion criteria may significantly influence conclusions. To the best of our knowledge, no prior systematic review or meta-analysis has specifically focused on studies of longitudinal AVG interventions targeting increases in PA behaviors.

**Objective:**

The aim of this study was to obtain insights into when and why longitudinal AVG interventions are more or less successful for sustained increases in PA, especially for public health.

**Methods:**

Six databases (PubMed, PsycINFO, SPORTDiscus, MEDLINE, Web of Science, and Google Scholar) were reviewed until December 31, 2020. This protocol was registered in the International Prospective Register of Systematic Reviews (PROSPERO: CRD42020204191). For inclusion, randomized controlled trials had to prominently (>50% of intervention) feature AVG technology, involve repeated AVG exposure, and target changes in PA behavior. Experimental designs had to include ≥2 within- or between-participant conditions with ≥10 participants per condition.

**Results:**

A total of 25 studies published in English between 1996 and 2020 were identified, with 19 studies providing sufficient data for inclusion in the meta-analysis. Our findings indicated that AVG interventions had a moderately positive effect, thereby increasing overall PA (Hedges *g*=0.525, 95% CI 0.322-0.728). Our analysis showed substantial heterogeneity (*I*^2^=87.7%; Q=154.1). The main findings were consistent across all subgroup analyses. The comparison between PA assessment type groups showed a moderate effect for objective measures (Hedges *g*=0.586, 95% CI 0.321-0.852) and a small effect for subjective measures (Hedges *g*=0.301, 95% CI 0.049-0.554) but no significant difference between the groups (*P*=.13). The platform subgroup analysis indicated a moderate effect for stepping devices (Hedges *g*=0.303, 95% CI 0.110-0.496), combination of handheld and body-sensing devices (Hedges *g*=0.512, 95% CI 0.288-0.736), and other devices (Hedges *g*=0.694, 95% CI 0.350-1.039). The type of control group showed a wide range of effects sizes, ranging from a small effect size (Hedges *g*=0.370, 95% CI 0.212-0.527) for the passive control group (nothing) to a moderate effect size for the conventional PA intervention group (Hedges *g*=0.693, 95% CI 0.107-1.279) and ultimately to a large effect size for sedentary game as control groups (Hedges *g*=0.932, 95% CI 0.043-1.821). There was no significant difference among the groups (*P*=.29).

**Conclusions:**

AVGs represent a promising tool for PA promotion among the general population and clinical subpopulations. However, significant variabilities in AVG quality, study design, and impact were also detected. Suggestions for improving AVG interventions and related research will be discussed.

**Trial Registration:**

PROSPERO CRD42020204191; https://www.crd.york.ac.uk/prospero/display_record.php?RecordID=204191

## Introduction

### Background

Over the last 50 years, video games have emerged as one of the most popular forms of global entertainment. Recent estimates are that 3.2 billion people played video games in 2022, with more player growth projected (+5.4% from 2020 to 2021, projected to reach 3.6 billion by 2025) [1]. The growing popularity of video games has presented public health stakeholders with opportunities for reaching and engaging billions of gamers in health interventions of various types, that is, games for health. Physical inactivity is an extremely prevalent and consequential behavioral risk factor; it is associated with a wide assortment of serious chronic physical illnesses (heart diseases, cancers, diabetes), poor mental health [2], and ultimately, mortality [3-5]. A 2012 study in The Lancet by Kohl et al [6] estimated that physical inactivity had become the fourth leading cause of death worldwide, characterizing this as a “pandemic of physical inactivity.” Yet, despite scientific consensus and significant investments in public health initiatives, rates of physical inactivity are currently not falling or are not stable but are rising globally [6-8].

A subset of video games for health with the potential for helping to mitigate this global public health challenge is collectively referred to as active video games (AVGs), that is, video games that encourage physical activity (PA). In some cases, these AVGs are explicitly designed to prioritize promoting PA; in other cases, AVGs are designed to prioritize other targets (eg, entertainment, profit) such that PA promotion is a secondary consideration. Collectively, AVGs involve images or text on a digital screen changing based on the sensor-detected arm, leg, or full-body movement. However, AVGs vary significantly in several respects. For example, digital screens alone include immersive head-mounted displays, external PC or television monitors, or smartphones. Movement can be detected using a variety of sensors, including wearable accelerometers, cameras, pressure-sensing pads and platforms, or combinations thereof. Sensor-detected movement can influence gameplay either synchronously (in real time) or asynchronously. AVGs are also thematically varied, including simulations of familiar sports (eg, boxing, tennis, golf) and dancing, as well as navigating novel fantasy worlds and scenarios.

Some researchers have used the term exergame, defining it either as synonymous or overlapping with an AVG. Those who define exergame more narrowly typically do so based on the traditional definition of exercise, that is, PA that is specifically intended to improve or maintain physical fitness with a planned, repetitive, or structured format [9-11]. However, as noted, others have explicitly expanded the definition of exergames to include all “interactive video gaming that stimulates an active, whole-body gaming experience” [12] or “videogames that require bodily movement to play and function as a form of physical activity” [13], equating AVGs and exergames. Here, we use the terms AVG and exergame interchangeably but default to using AVG when possible, for clarity. Overall, AVGs constitute an important portion of the global virtual fitness market and are predicted to generate a revenue of US $59,650.30 million by 2027 at a compound annual growth rate of 33.5% [14]. So far, AVGs have most frequently been designed and marketed to consumers as forms of entertainment as opposed to health care. Nevertheless, behavioral health researchers have also used commercially available AVGs as major components of treatment interventions designed to promote PA and other clinically meaningful outcomes, sometimes testing these interventions by using randomized controlled trials. A recent content analysis of AVGs used in randomized controlled trials found that 72% of those AVGs were developed by for-profit game studios for the commercial entertainment market; only 13.3% were developed using government or foundation grant funding for health research and applications, with AVGs designed for the Nintendo Wii and Microsoft Xbox Kinect platforms best represented [15]. Similar trends were found with games for health research in general, where around 60% of the games used in these research projects used commercially available games [16].

### AVGs and Public Health: Opportunities and Concerns

As AVGs have grown in popularity, public health stakeholders have alternatively considered both opportunities and concerns. Notably, the American College of Sports Medicine has referred to AVGs as “the future of fitness” [10], and many reviews express optimism about AVGs’ potential for increasing PA in children and adolescents especially [17]. Yet, concerns have also been raised about the potential for AVGs to sustain long-term engagement or sufficiently intense levels of PA to achieve a meaningful public health impact [18]. A balanced 2018 review of exergaming for children and adolescents by Benzing and Schmidt [10] noted these opportunities and concerns, concluding that so far, AVGs’ “potential is frequently underexploited,” and we noted that many of the issues explored in depth by Benzing and Schmidt [10] apply to adults as well.

### Past Systematic Reviews of AVG Intervention Research

Over the past decade, a number of outcome-focused systematic reviews and meta-analyses related to AVG research have been published covering a wide spectrum of target populations and health conditions, including PA, weight loss, motor skills, rehabilitation, and physical education [16,17,19,20]. A 2022 protocol for an AVG review by Hoffmann and Wiemeyer [19] pointed out that “most studies focus on specific training effects or specific target groups” and that “a comprehensive summary of…effects with exergames in healthy adults is still missing.” Healthy adults have often been excluded from the meta-analytic reviews of AVG effects. Indeed, several meta-analyses that have focused on changes in PA as the focal outcome have restricted inclusion to specific subpopulations, often with respect to a chronic health condition or limited age range. For example, a number of systematic reviews of AVG studies have exclusively focused on children and young adults [21-24]. Among these, several focused their attention further still on children with specific health conditions, for example, children with a developmental coordination disorder [23] or children with autism spectrum disorder [25]. Another recent systematic review by Garcia-Agundez et al [26] focused exclusively on AVGs for adults in rehabilitation for Parkinson disease. Simmich et al [27] conducted a systematic review and meta-analysis on AVGs for those in rehabilitation for respiratory conditions, and Smits-Engelsman et al [28] performed a systematic review and meta-analysis that included AVGs for developmental coordination disorders. Other systematic reviews focused just on AVGs used by geriatric or older adults, targeting general PA [20] or AVGs for condition-specific treatments, for example, van Santen et al [29] completed a systematic review of AVGs for older adults with dementia and Tahmosybayat et al [30] did a systematic review of AVGs for older adults by targeting postural control. A rare systematic review that focused on studies of AVGs targeting energy expenditure among healthy adults was conducted by Dutta and Pereira [31]. Their meta-analysis found increased energy expenditure while playing AVGs, included 15 studies, and was published nearly 8 years ago in 2015. In addition, previous reviews and meta-analyses involving AVGs tended to focus on a limited set of AVGs and AVG platforms or conflated multiple AVG platforms. For example, while the labels AVG and exergame can be used interchangeably [32,33], some authors have employed different terms such as virtual reality [34] or interactive computer gaming [30] to refer to AVGs. Such naming inconsistency could lead to identical studies reviewed under both AVG and exergame categorizations. Moving forward, we would like to propose the term “active video games” as an umbrella term encompassing both AVGs and active virtual reality and any digital game that requires players’ upper-, lower-, or full-body movement as part of the play. We will also consider all active video gaming platforms to ensure maximum coverage.

Several concerns have been raised related to using AVGs in behavioral health interventions, specifically interventions targeting PA. For example, some AVGs might inadvertently encourage deception of motion-sensing systems (eg, if participants replace full-body movements with small hand gestures to maximize game-specific outcomes like score). This concern was partially mitigated by meta-analyses conducted by Dutta and Pereira [31] and Peng et al [35]. Both meta-analyses showed that energy expenditure while playing AVGs was comparable to that while performing traditional physical activities and was elevated relative to sedentary games; however, Peng et al [35] found that AVGs that used lower body and full-body movement-sensing systems produced more energy expenditure than AVGs that used upper body (ie, handheld) movement-sensing systems. Critics have also questioned whether AVG interventions might fail to promote, or worse, inhibit PA outside of game play. Focusing our systematic review and meta-analysis on only AVG interventions that have used longitudinal designs to target and assess PA behaviors with well-validated objective and subjective measures can help address those concerns.

### Objectives of This Review

Given the massive scale of the global video game industry, the supporting technology and content of AVGs is rapidly evolving. As such, frequently updated systematic reviews and meta-analyses are important tools for helping stakeholders understand what is known about AVGs. Specifically, an updated overview of AVG intervention trials targeting PA, including both clinical and general populations, would fill a gap in the literature. Therefore, we conducted a systematic review and meta-analysis focused on the following research questions related to AVG intervention trials targeting PA behaviors:

1. What samples and settings were used in these AVG intervention trials?
2. What was the risk of bias in these studies?
3. What was the overall effect of these AVG interventions on postintervention PA?
4. Were there subgroups among these AVG intervention trials associated with more or less postintervention PA? Specific subgroups of interest include (1) PA assessment method (objective vs subjective measures of PA), (2) aspects of the AVG platform’s motion-sensing system, and (3) comparison groups used (nothing, a conventional PA intervention, or a sedentary video game).

## Methods

### Design

To provide an updated and comprehensive systematic review and meta-analysis, we performed this research in accordance with the PRISMA (Preferred Reporting Items for Systematic Reviews and Meta-Analyses) guidelines [1]. The protocol of this review was registered in the International Prospective Register of Systematic Reviews (PROSPERO: CRD42020204191).

### Selection Criteria

The inclusion and exclusion criteria are summarized in Table 1.

**Table 1:** Inclusion and exclusion criteria for the intervention studies in this review.

Criteria	Inclusion criteria	Exclusion criteria
Paper type	Original and published in peer-reviewed journals or full-length conference proceedings	Secondary reports, reports that were not peer-reviewed
Language	Only English language reports were eligible, study materials (survey and interviews) could be in any language	Secondary reports, reports that were not peer-reviewed
AVG^a^ qualities	Games must be interactive and digital, that is, powered by electricity	Digital media that was not interactive (eg, exercise videos), board games
Physical activity required by the AVG	AVGs must require gross motor movements that went beyond mere finger movements with the goal to enhance, maintain, or regain health	Interactive games that could be played exclusively by making small finger movements (eg, sedentary video games)
AVG intervention qualities	Interventions had AVGs as the sole or primary part of treatment (≥50% of the intervention)	Interventions that featured AVGs as a secondary aspect of treatment (<50%) or that made playing AVG optional
Study design	Random assignment to one of at least 2 conditions, within- or between-participants; at least 10 participants per condition; control conditions included either conventional physical activity intervention, sedentary games, or a passive group (nothing/waitlist); repeated (≥2) exposures to AVG(s)	Correlational studies, 1 group pre/posttest or 1 group posttest-only designs, qualitative studies, studies that involved just 1-time exposure to AVG(s)

^a^AVG: active video game.

### Primary Outcome

The primary outcome considered in this meta-analysis was PA. PA is all bodily movements produced by contracting skeletal muscles that substantially increase energy expenditure and can be represented by direct assessments of physical movement (eg, using one or more accelerometers, GPS technology) or validated questionnaires (eg, Physical Activity Recall, Yale Physical Activity Survey, Godin-Shephard Leisure-Time Physical Activity Exercise Questionnaire) [36]. Postintervention PA was used as the primary outcome for this meta-analysis rather than preintervention-to-postintervention changes in PA for several reasons. First, not all the AVG intervention studies included in our meta-analysis reported preintervention PA, but all studies included postintervention PA. Second, some researchers have suggested that pre-post effect sizes should not be used in meta-analyses [37]. A methodological review of meta-analyses by Rubio-Aparicio et al [38] found that the majority of meta-analyses (76%) used postintervention scores exclusively and recommended this as the preferred option for meta-analysis.

### Search Strategy

Between March 1, 2020, and December 31, 2020, we searched each of the following 6 electronic databases: PubMed, EBSCO (Elton B Stephens Company; PsycINFO, SPORTDiscus, MEDLINE), Web of Science, and Google Scholar for relevant studies. The phase I of the search was limited to English, human-related, and peer-reviewed synthesis papers (types: review, narrative review, systematic review, meta-analysis, and synthesis of synthesis paper) published by April 30, 2020, in the Google Scholar or PubMed databases. For the synthesis of synthesis papers, we first extracted separate synthesis papers and then examined the individual original papers from each of the separate synthesis papers. This uncovered 201 nonduplicated publications. We extracted 3724 individual original papers from these synthesis papers.

**Figure 1:**
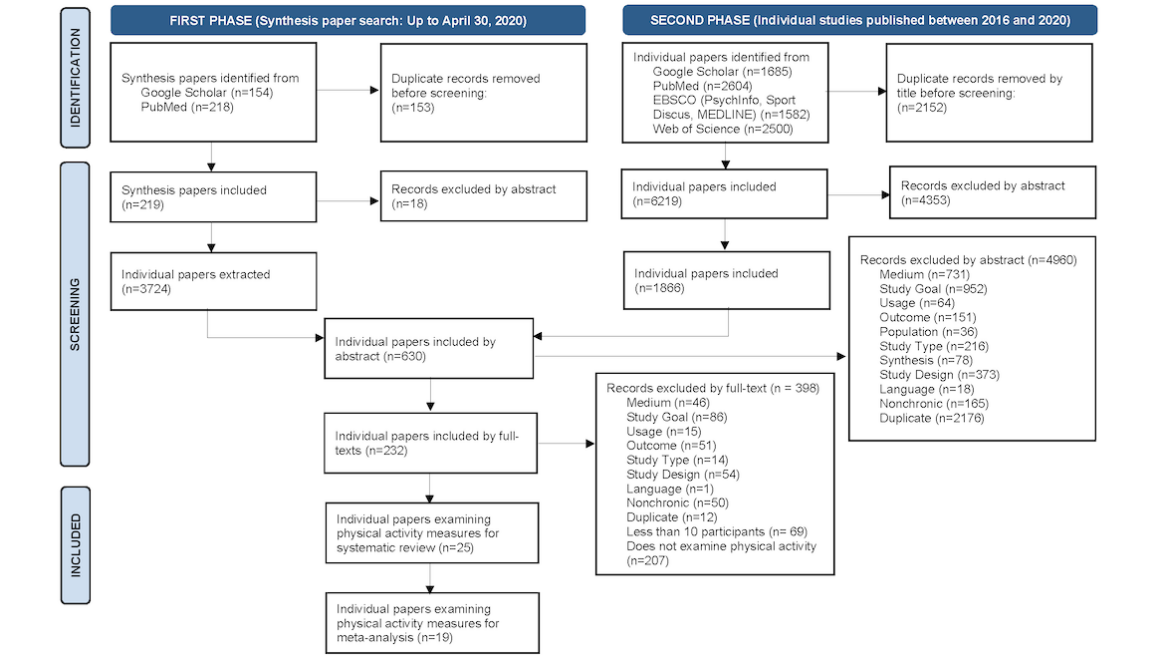
PRISMA (Preferred Reporting Items for Systematic Reviews and Meta-Analyses) flow diagram. EBSCO: Elton B Stephens Company.

In phase II, we searched for individual original studies published between January 1, 2016, and December 31, 2020, to ensure that more recent studies not included in the synthesis papers were included. The same 6 electronic databases, that is, PubMed, EBSCO (PsycINFO, SPORTDiscus, MEDLINE), Web of Science, and Google Scholar, were included in our individual paper search. We found 1866 individual papers using the keyword search. We then merged the search results in both phases and identified 630 unique papers by abstract and continued to read the full text of these papers. This full-text reading resulted in 232 unique publications targeting a range of outcomes. We further identified 25 unique publications targeting PA measures for our systematic review, and 19 publications included sufficient data from PA measures for inclusion in our meta-analysis. A summary of this 2-phase search process is illustrated in Figure 1, which shows the PRISMA flow diagram. The Boolean search phrases that were used for both phases are listed in Multimedia Appendix 1.

### Data Extraction and Content Coding

Three independent reviewers (DA, KJL, and CVS) with different academic backgrounds (Biology, Health, and Physical Education, respectively) participated in paper selection and data extraction to ensure fair and comprehensive coverage. They received 2 training sessions per week throughout the 4-month project preparation period from May to September of 2020. The interrater reliability was assessed every week to ensure that it was consistently higher than 85% during the training and was maintained at over 93% for the rest of the coding process. Differences were solved through discussion till all coders agreed on how to proceed. Employing a standardized data extraction sheet, they recorded the information about the characteristics of each study, its participants, the intervention, and the outcome measures. Authors were contacted using a standardized email template when their publications did not contain all the information listed above.

### Evaluation of Study Quality

The quality of the included papers was assessed by implementing GRADE (Grading of Recommendations Assessment, Development and Evaluation) [39]. The 6 components evaluated consisted of random assignment (to avoid selection bias), allocation concealment (to avoid selection bias), blinding of participants and personnel (to avoid performance bias), blinding of outcome assessment (to avoid detection bias), completeness of reporting of some outcome data (to avoid attrition bias), and selectivity in reporting (to avoid reporting bias). We added a seventh criterion, which assessed any potential adjustment for additional confounding variables. Each category was given a score from –1 to +1, where –1 represented high risk of bias, 0 represented unclear risk, and +1 represented low risk of bias. The overall score was an average of each individual component on the –1 to +1 scale. No papers were excluded based on the GRADE quality assessment. The same 3 independent reviewers conducted the evaluation following the same protocol detailed in the previous section. More specifically, the interrater reliability was consistently maintained at over 93% for the coding process after the training sessions, and differences were solved through discussion.

### Statistical Analysis

Statistical heterogeneity between studies was quantified using the *I^2^* statistic, which describes the percentage of variation across studies due to heterogeneity rather than sampling error or chance (0%-40%: negligible heterogeneity; 30%-60%: moderate heterogeneity; 50%-90%: substantial heterogeneity; 75%-100%: considerable heterogeneity) [40]. The level of significance was set at *P*<.05. Each outcome was combined and calculated using the MS Office Excel macro sheets provided by Borenstein et al [41]. Statistical analyses were conducted with the Comprehensive Meta-Analysis 3.0 (BioStat Inc) software package.

### Meta-analytic Procedure

For the purpose of this meta-analysis, the study designs were homogenized by only using postintervention measures. Parameters in the same category within a study were pooled with a fixed-effects model. The meta-analyses used a random-effects model with sufficient homogeneity in terms of design and comparators. The continuous outcomes were converted into standardized mean differences with 95% CI. The standardized mean difference was the effect size measure for calculating Hedges *g*. The Hedges *g* metric is generally preferred to Cohen *d* because it has better small sample properties and better properties when the sample sizes are significantly different from each other. Our meta-analytical procedures followed the methods outlined by Borenstein et al [41]. An independent meta-analytical model was applied to each subgroup analysis. The subgroups were categorical moderators in each model and were defined based on the characteristics of the individual studies: assessment type (eg, objective vs subjective measures of PA), platform (eg, handheld device such as Nintendo Wii, full-body sensing device such as Microsoft Xbox with Kinect, a combination of handheld device and full-body sensing device), and type of control group (passive control/nothing, conventional PA intervention, or sedentary videogames). In order to determine the risk of bias in the individual studies, Egger test was applied, and the funnel plot symmetry/asymmetry was assessed visually. The significance level for Egger regression was set at *P*<.01, as previously suggested [42].

## Results

### Samples and Settings

After examining the outcomes of the 232 papers that met the general inclusion criteria, 25 studies were identified that reported 1 or more PA outcomes. Of those, 19 studies provided sufficient information to be eligible for the meta-analysis. Figure 1 shows the flow diagram of the search and study selection; the checklist can be found in Multimedia Appendix 2. Multimedia Appendix 3 summarizes the details of the intervention and AVG characteristics, including intervention session lengths, frequencies, total length, AVG platform, specific AVG game titles, control group descriptions, PA assessment method(s), and metrics. Multiple AVG interventions offered participants more than one AVG platform (6/25, 24%); the most popular platforms used were Nintendo Wii (11/25, 44%) and Kinect for Xbox 360 (9/25, 36%).

### Participant Details

The locations of the 25 PA interventions were distributed across 4 regions: North America (14/25, 56%), Europe (4/25, 16%), Asia (4/25, 16%), and Oceania (3/25, 12%). The top 4 countries included the United States of America (12/25, 48%), Australia (2/25, 8%), Canada (2/25, 8%), and Singapore (2/25, 8%); 7 countries contributed 1 study each (China, Finland, Ireland, New Zealand, Spain, Turkey, and the United Kingdom). The total enrollment across all 25 studies was 2888 participants (mean_total_ 115.52, SD 217.30; range 20-1112; mean_experiment_ 55.00, SD 107.49; range 10-557; mean_control_ 60.52, SD 110.89; range 10-555). Although the average age of the participants was 18.61 years, the studies involved participants in a wide range of ages (mean_range_ 4-71 years, mean_SD_ 20.00 years): 72% (18/25) of the studies included children and adolescents (0-18 years), 16% (4/25) of the studies included adults (18-64 years), and 12% (3/25) of the studies included both adults and older adults (>18 years). The average percentage of men across all studies was 50.71% (SD 17.94%; range 0%-81.25%). 

A total of 22 (88%) studies conducted pretest and posttest assessments of PA, and only 3 studies conducted posttest assessments (with no pretest). Of the 25 studies, 12 (48%) included an inert control group, 9 (36%) included a nonequivalent but active comparison group that encouraged PA, 3 (12%) included sedentary game play as the control group, and 1 (4%) included both an active comparison group and a sedentary health education control group. Of the 25 studies, 23 (92%) were conducted in a field setting, with only 1 (4%) performed in a laboratory setting; 1 (4%) study did not specify the setting. Most field studies were conducted in schools (8/23, 32%) or at home (7/23, 28%); other field settings included hospitals (n=2), medical centers (n=2), group living facilities (n=2), physical therapy practice (n=1), or were unclearly reported (n=1). Of the 25 studies, 18 (72%) reported no specific medical conditions for the participants. The rest 7 studies were conducted with clinical populations with special conditions: overweight/obesity (2/25, 8%), cancer (1/25, 4%), stroke (1/25, 4%), and others (3/25, 12%).

Nintendo Wii and Microsoft Xbox with Kinect were the exergame platforms used in most of the 25 studies: Wii was used as the sole platform in 7 (28%) studies, Xbox 360 with Kinect was used as the sole platform in 5 (20%) studies, and together, they were used as joint platforms in another 4 (16%) studies. They were followed by PlayStation (4/25, 16%), PCs (2/25, 8%), and the LeapTV platform (1/25, 4%). No study used immersive virtual reality that required a headset.

### Risk of Bias in Individual Studies

**Figure 2:**
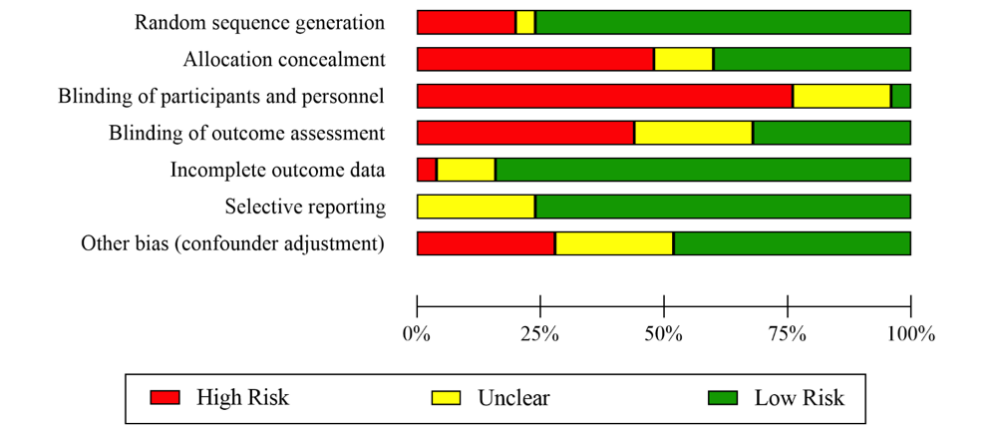
Study quality according to GRADE (Grading of Recommendations Assessment, Development and Evaluation) tool.

The overall study quality GRADE was calculated by averaging the scores for the 7 components of study quality on a –1 to +1 scale (–1=low quality; +1=high quality). The distribution of the GRADE scores on all 7 components is summarized in Figure 2. Overall, the mean total GRADE score tended to be relatively low at –0.20 (SD 0.37; range –0.71 to +0.43). Overall, the GRADE score and the 7 GRADE subscores are reported for all studies individually in Multimedia Appendix 4. The top 3 high-risk factors for bias were the lack of blinding of participants and personnel (19/25, 76%), lack of allocation concealment (12/25, 48%), and lack of blinding of outcome assessment (11/25, 44%). Conversely, the top 3 factors indicating higher quality or low risk of bias were completeness in reporting outcome data (21/25, 84%), random assignment (19/25, 76%), and avoiding selective reporting (19/25, 76%). The mean total GRADE score was then correlated with the year of publication and other study descriptors such as the continent where the study was conducted, participant age, and idealized and actual intervention duration. The total GRADE score was negatively correlated with participant age (*r=–*0.48, 95% CI *–*0.74 to *–*0.05; *P=*.03; N=25) and idealized intervention duration (*r=*0.41, 95% CI 0.00 to *–*0.69; *P*<.05; N=25), meaning that interventions targeting younger participants and with shorter intervention durations were associated with higher publication quality. All other correlations were nonsignificant (*P*=.06-.71).

### Meta-analysis

**Figure 3:**
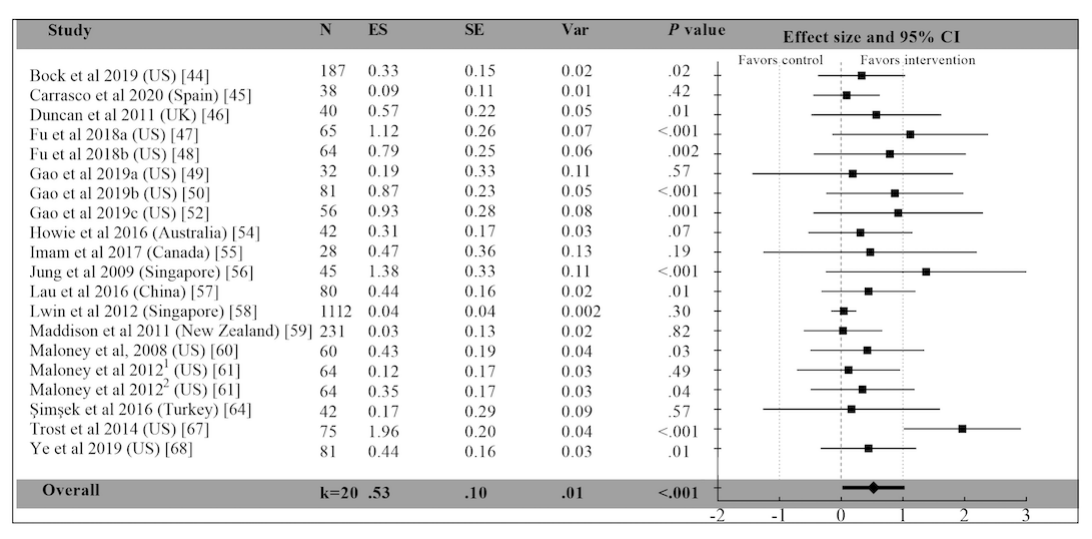
Forest plot of the standardized mean effect sizes of the individual studies [44-68] after active video game intervention. The study of Maloney et al (2012) appears twice to reflect separate calculations for subjective and objective measures of physical activity. Squares represent effect sizes for individual studies, diamonds (in bold) represent the average of effect sizes from individual studies. ES: effect size (Hedges g); N: number of participants; Var: variance.

A total of 20 PA outcomes were available in 19 studies after within-study pooling. All objective or subjective PA measures were pooled within each study. One study employing both subjective and objective PA measures was calculated twice. Overall results showed a significant effect in favor of the intervention (Hedges *g*=0.525, 95% CI 0.322-0.728). See Figure 3 [43-68] for details. This analysis showed substantial heterogeneity (*I*^2^=87.7%; Q=154.1).

### Subgroup Analysis

**Figure 4:**
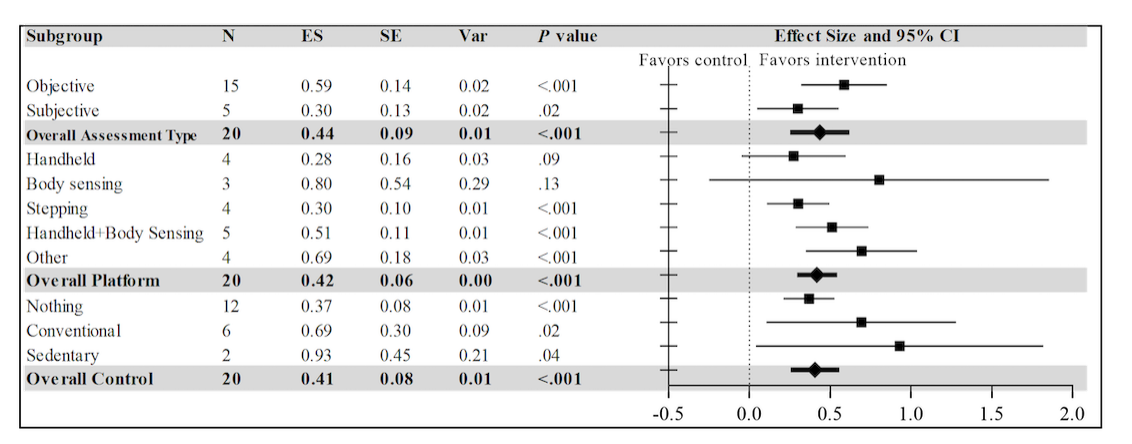
Forest plot of the standardized mean effect sizes of the subgroups. Subgroup analyses were performed with assessment type, platform, and type of control group as independent moderators. Diamonds (in bold) represent the average of individual effect sizes for subgroups within a given moderator category, and squares represent individual effect sizes for subgroups within a given moderator category. ES: effect size (Hedges g); N: number of participants; Var: variance.

Significant subgroup effects were found in almost all models (*P*<.001). The comparison between assessment type groups showed a moderate effect for objective measures (Hedges *g*=0.586, 95% CI 0.321-0.852) and a small effect for subjective measures (Hedges *g*=0.301, 95% CI 0.049-0.554) but no significant difference between the groups (*P*=.13). For platform groups, we compared 5 types of platforms based on how players interact with the consoles: handheld devices (eg, Wiimote), body-sensing devices (eg, Kinect), stepping devices (eg, dance pad), a combination of handheld and body-sensing devices (eg, Wii, Kinect), and other devices (eg, Leap television console). Results indicated a moderate effect for stepping devices (Hedges *g*=0.303, 95% CI 0.110-0.496), combination of handheld and body-sensing devices (Hedges *g*=0.512, 95% CI 0.288-0.736), and other devices (Hedges *g*=0.694, 95% CI 0.350-1.039). Neither the handheld nor the body-sensing devices were found to be statistically significant. In addition, no significant difference was found between the platform groups (*P*=.22). For type of control group, passive control (nothing), conventional PA intervention, and sedentary videogames were compared. The type of control group showed a wide range of effect sizes, with a small effect size for passive control group (nothing) (Hedges *g*=0.370, 95% CI 0.212-0.527), moderate effect size for conventional PA intervention group (Hedges *g*=0.693, 95% CI 0.107-1.279), and a large effect size for sedentary game as control groups (Hedges *g*=0.932, 95% CI 0.043-1.821). There was no significant difference among the groups (*P*=.29) (see Figure 4).

### Risk of Publication Bias

**Figure 5:**
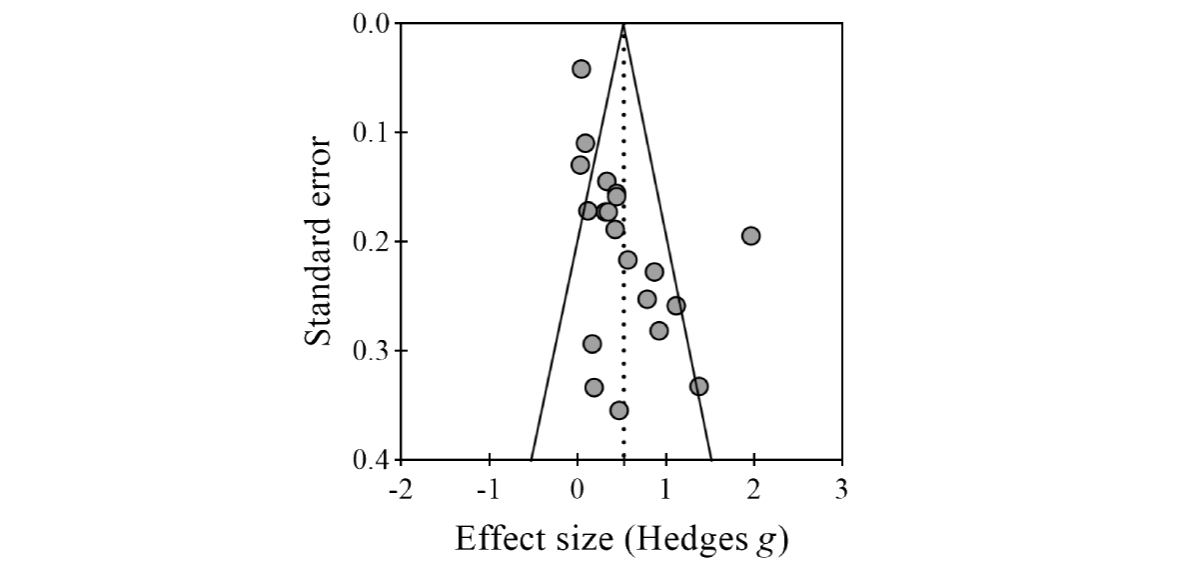
Funnel plot for publication bias assessment.

The Egger regression intercept showed a significant chance of publication bias (*P*=.001) although the funnel plot visually showed an asymmetric distribution of the studies (see Figure 5). Therefore, a series of in-depth analyses further scrutinized the level of significance as a way to understand the degree of publication bias. As reported above, the 25 studies (19 included in the meta-analysis) contained 53 variables related to PA, with an average of 2.12 variables per paper. Of these 53 variables, 19 (36%) showed significant improvements as a result of the AVG intervention, 33 (63%) did not improve significantly, and 1 (2%) PA outcome actually deteriorated, though it is worth mentioning that the measure was light PA and was expected to decrease (the study intended to increase moderate to vigorous PA and indeed observed the significant increase). When these findings were calculated within each of the 25 studies, 12 (48%) papers reported uniformly positive effects on PA, 0 (0%) reported uniformly negative effects, 10 (40%) reported nonsignificant effects, and 3 (12%) reported mixed effects.

## Discussion

### Principal Findings

Our systematic review identified 25 AVG intervention studies involving longitudinal AVG exposures that reported 1 or more PA outcomes and 19 studies that provided sufficient information to be eligible for the meta-analysis. Multiple AVG interventions offered participants more than 1 AVG platform, the most popular platforms being the Nintendo Wii (11/25, 44%) and Kinect for Xbox 360 (9/25, 36%). The average age of the participants was 18.61 years, but the studies involved participants in a wide range of ages, with 72% (18/25) of the studies targeting children and adolescents (0-18 years). Approximately 92% (23/25) of the studies were conducted in a field setting, with only 1 (4%) performed in a laboratory setting. Overall, study quality (mean total GRADE score) tended to be relatively low, a finding that limits generalizability and informs the need for higher study quality in future AVG intervention research. The top 3 high-risk factors for bias were the lack of blinding of participants and personnel (19/25, 76%), lack of allocation concealment (12/25, 48%), and lack of blinding of outcome assessment (11/25, 44%). Overall, our meta-analysis found that AVG interventions involving repeated exposure and that targeting PA had a moderate positive effect on increasing PA when compared to all control groups. This effect was stronger when PA was objectively measured (Hedges *g*=0.586) relative to when subjectively measured (Hedges *g*=0.301), though heterogeneity between studies made this difference not statistically significant. One interpretation for the larger effect sizes observed when PA was objectively measured involves the possibility that PA may have systematically been underreported when assessed with subjective self-report measures in the AVG intervention conditions specifically. Although, in general, research has shown that subjective measures of PA tend to be inflated relative to objective measures [69], intrinsic motivation and flow states associated with AVGs are associated with temporal distortion [70] and less discomfort [71], which may contribute to lower self-reported estimates of PA during AVG interventions relative to control groups.

The platform analysis indicated that AVGs employing handheld devices (eg, Nintendo Wii) (Hedges *g*=0.275; *P*=.09) and body-sensing devices (eg, Microsoft Xbox with Kinect) (Hedges *g*=0.804; *P*=.13) produced a wide range of effect sizes that were not statistically average significant; by contrast, AVGs employing a combination of both handheld and body-sensing devices produced a significantly average effect (Hedges *g*=0.512; *P*<.001). However, AVGs that employed stepping devices (eg, haptic dance pad), when used alone, did produce a significant average effect (Hedges *g*=0.303; *P*=.002). This is consistent with findings from an earlier 2011 meta-analysis of AVG effects on PA assessed during game play, which found that AVGs that used lower body and full-body movement-sensing systems produced more energy expenditure than AVGs that used upper body (ie, handheld) movement-sensing systems [35]. Individual studies evaluating AVGs employing different movement-sensing systems have assessed energy expenditure in different ways with different samples and have reported mixed results. For example, a study by Miyachi et al [72] estimated steady state energy expenditure assessed in an open-circuit indirect metabolic chamber while playing 68 different AVG activities on the Wii Sports (n=5) and Wii Fit Plus (n=63) platforms. Wii Sports activities use handheld-only motion sensing, while Wii Fit Plus activities use a combination of a handheld device and a stepping device (a Wii balance board). Overall, 46 (67%) activities were classified as light intensity (<3 metabolic equivalents of task [METs]), 22 (33%) activities as moderate intensity (3.0-6.0 METs), and none as vigorous intensity activities (>6.0 METs). Playing all 5 Wii Sports AVGs was associated with light-to-moderate intensity PA, with METs ranging from 2.0 to 4.2: golf (2.0), bowling (2.7), baseball (3.0), tennis (3.0), and boxing (4.2). A complementary study by Bosch et al [43] assessed the heart rates among 20 young adults (age range 23-27 years) while playing Wii Sports Boxing (handheld-only motion sensing) for 30 minutes relative to their maximum heart rate (individually calibrated using a treadmill test). Participants’ mean heart rate response while playing Wii Sports Boxing was 143 bpm, or 75.5% of their maximum heart rates. However, the mean heart rate response for experienced players was significantly lower than that for inexperienced players. These findings and our results support the assertion that certain AVGs employing handheld-only motion systems can still produce light-to-moderate intensity PA in some samples under some circumstances. All else equal, AVGs employing multiple motion-detecting systems tend to produce higher intensity PA more consistently. However, aspects of the AVG itself (eg, Wii Sports Golf vs Wii Sports Boxing) often explain more variance in PA intensity than the platform’s motion-sensing system (eg, handheld-only vs multiple body sensing devices; Wii Sports vs Wii Fit Plus platforms) [72].

The type of control group was related to the differential effects on PA, with a smaller effect found for passive control groups (nothing), a moderate effect found for conventional PA intervention, and a larger effect found when a sedentary game was used as a comparison group. These findings suggest that AVGs can be more than just entertainment, promoting PA in diverse subpopulations around the world, regardless of whether the games were designed for entertainment or for behavioral health. Indeed, most of the devices are commercially available consumer products. Additionally, over 90% of the intervention studies included in our review were conducted in the field. All of these suggest AVG’s high potential for public health impact. In terms of informing future research on AVG interventions, findings suggest that study design decisions, including selection of PA measures, platform selection, and control/comparison groups, may contribute to predicting anticipated effect sizes—an important consideration for a priori power analysis and determination of sample size. More specifically, while participants’ age ranged widely across the studies (4-71 years), the average age was still toward the younger end (around 18 years), suggesting that AVGs might still be primarily applied among children and adolescents. The study quality seemed to be better when the participants were younger and when the ideal intervention duration was longer. Most of the participants in the studies (>70%) were generally healthy participants without particular health conditions. Future studies might want to expand to additional age groups and clinical populations with improved research rigor to better investigate the AVG’s effect for PA promotion across the developmental as well as the health spectrum.

### Limitations in This Review

Several limitations of this systematic review and meta-analysis are worth noting. First, our review shows that only a small fraction of empirical studies investigating AVGs involve a combination of repeated exposures to the game in the context of an intervention that targeted changes in PA as the primary outcome. Of the 232 papers that met the general inclusion criteria in our review, only 25 studies were identified that reported 1 or more PA outcomes, and of those, only 19 studies provided sufficient information to be eligible for the meta-analysis. Furthermore, demographic content analysis of the samples revealed that although studies included participants from a wide range of ages (mean_range_ 4-71 years), a majority (72%) of the studies focused on children and adolescents (0-18 years), thereby limiting the scope and generalizability in the sense that the average effect sizes estimated across all AVG intervention studies may apply more to children and adolescents than older age groups. The time required to conduct an analysis and write up the findings limited inclusion to studies published from 1996 to December 31, 2020; if any repeated-exposure AVG intervention studies targeting PA were published since, they were not included, limiting the scope of our review. As AVG technology is continuously advancing, PA interventions incorporating newer AVGs may be more effective at promoting PA; however, testing this hypothesis was beyond the scope of this meta-analysis. The overall study quality was low, with high heterogeneity in quality among the studies. Future AVG studies should include more comprehensive and higher quality measures of PA behavior as well as improve the overall study quality. Similarly, as the number of high-quality published AVG intervention studies focused on changes in PA increase, future meta-analyses will be better powered to conduct a greater range of subgroup analyses (eg, comparing immersive vs nonimmersive AVGs, AVGs that include or exclude specific motivation and behavior change techniques and theories [73]). Last but not the least, around half of the studies found significant improvement in the outcome measures; therefore, publication bias could not be ruled out. The combination of risk of bias and publication bias reduces the overall quality of evidence in the AVG intervention literature to either low or very low. As such, as a field, AVG intervention researchers should aim for increasing methodological rigor and more balanced reporting of findings to obtain realistic insights into the efficacy of AVG interventions, especially those employing longitudinal designs and tracking PA in the field, including PA engaged in outside of game play. It is noteworthy that this work builds on prior systematic reviews and meta-analyses of AVG research, in particular, Peng et al [35] and Dutta and Pereira [31], which focused on energy expenditure among healthy adults while playing AVGs.

### Conclusion

In conclusion, our review represents an important contribution toward synthesizing PA intervention research that centers on exergames or AVGs. As more sophisticated AVGs become increasingly available and affordable to more people—a trend driven largely by the video games for entertainment industry—we expect to see an increasing proportion of all PA interventions incorporating this technology. In light of this, PA interventionists and other public health stakeholders would do well to follow the development of AVG technologies and to understand how and when they can be leveraged for maximum public health impact.

## Appendix

Multimedia Appendix 1 Boolean search phrases.

Multimedia Appendix 2 PRISMA (Preferred Reporting Items for Systematic Reviews and Meta-Analyses) 2020 item checklist.

Multimedia Appendix 3 Sample and intervention characteristics with outcomes for 25 studies of active video game interventions targeting physical activity.

Multimedia Appendix 4 GRADE (Grading of Recommendations Assessment, Development and Evaluation) scores.

## Abbreviations

AVG: active video game
EBSCO: Elton B Stephens Company
GRADE: Grading of Recommendations Assessment, Development and Evaluation
MET: metabolic equivalent of task
PA: physical activity
PRISMA: Preferred Reporting Items for Systematic Reviews and Meta-Analyses
PROSPERO: International Prospective Register of Systematic Reviews
