# Supplementary material for: Active Video Game Interventions Targeting Physical Activity Behaviors: Systematic Review and Meta-analysis
Source: J Med Internet Res. 2023 May 16;25:e45243. doi: 10.2196/45243 (PMC10230359; doi:10.2196/45243)
Supplement: Multimedia Appendix 1 [file jmir_v25i1e45243_app1.docx]

**Phase I: Synthesis Article Search**

**Databases: Google Scholar/PubMed**

Google Scholar:

Active (video) gam* meta analysis OR Active (video) gam* systematic review

Exergam* meta analysis OR exergam* systematic review

Virtual reality AND (systematic review OR meta-analysis OR meta analysis)

Wii OR Kinect OR Xbox OR Eye Toy OR Dance dance revolution OR Nintendo switch) AND (Systematic review OR Meta-analysis OR Meta analysis)

Return articles dated between: * - 2020

PubMed:

Active (video) gam* meta analysis OR Active (video) gam* systematic review

Exergam* meta analysis OR exergam* systematic review

Virtual reality[title/abstract]

Wii[title/abstract] OR Kinect[title/abstract] OR Xbox[title/abstract] OR Eye Toy[title/abstract] OR Dance dance revolution[title/abstract] OR Nintendo switch[title/abstract]

Results by Year: * - 2020

Article Type: Review OR Systematic Review

**Phase II: Individual Article Search**

**Databases: Google Scholar/PubMed/EBSCO/Web of Science**

Google Scholar:

Advanced Search

With all of the words: Active video games Exergames Virtual reality Health Virtual reality rehabilitation

Without the words: Review Meta Analysis

Return articles dated between: 2016-2020

PubMed:

Title/Abstract: Active (video) gam* OR Exergam* OR virtual reality OR Wii OR Kinect OR Xbox OR

Eye Toy OR Dance Dance Revolution OR Nintendo

Results by Year: 2016-2020

Article Type: Clinical Trial OR Randomized Clinical Trial

EBSCO:

Selected Databases: APA PsycInfo, SPORTDiscus with Full Text, MEDLINE

(Acti* OR Interactive OR Computer OR Video OR Exer OR Serious OR (Virtual AND Reality) OR VR) AND Gam* OR Wii OR Kinect OR Nintendo OR Xbox OR exergames OR exergaming OR “active video game” OR “active videogaming”

From: 2016 to 2020

Language: English

Web of Science:

((TI=(Exergam OR Active video gam OR Virtual reality OR Wii OR Kinect OR Xbox OR Eye Toy OR Dance dance revolution OR Nintendo NOT review NOT meta analysis))) AND LANGUAGE: (English) AND DOCUMENT TYPES: (Article)

Refined by: DOCUMENT TYPES: (ARTICLE OR PROCEEDINGS PAPER OR EARLY ACCESS OR BOOK CHAPTER )

Publication Date: 2016-2020

Indexes: SCI-EXPANDED, CPCI-S, BKCI-S.
