# Supplementary material for: Active Video Game Interventions Targeting Physical Activity Behaviors: Systematic Review and Meta-analysis
Source: J Med Internet Res. 2023 May 16;25:e45243. doi: 10.2196/45243 (PMC10230359; doi:10.2196/45243)
Supplement: Multimedia Appendix 3 [file jmir_v25i1e45243_app3.docx]

**Table: Sample and Intervention Characteristics with Outcomes for 25 studies of AVG Interventions Targeting PA**

|  | **Sample characteristics** | | | | **Intervention** | | | | | **Assessment method** | **Outcomes** |
| --- | --- | --- | --- | --- | --- | --- | --- | --- | --- | --- | --- |
| **Study (Country)** | **E (n)** | **C (n)** | **Men %** | **Condition** | **Session length (min)/ frequency (session/wk)** | **Total length (min/weeks)** | **Platform** | **Game/Activity** | **Control** |  |  |
| Bock B. C., et al., 2019 (USA) [44] | 93 | 190 | 21 | NSC | 50 / 3 | 1800 / 12 | Wii and Kinect for Xbox 360 | Your Shape Fitness Evolved / Kinect Adventures / Zumba / Kinect Sports / The Biggest Loser | Standard aerobic exercise or weekly mailed health and wellness materials | Physical Activity Recall (S) | MVPA minutes/ week (⭡) |
| Carrasco, M., et al., 2020 (Spain) [45] | 22 | 16 | 32 | NSC | 60 / 2 | 720 / 6 | Wii (U) | Tennis / baseball / bowling / boxing | Nothing | Yale Physical Activity Survey (S) | YPAS-TTI (⭤ )  YPAS TEEI (⭤ )  YPAS VI (⭤ )  YPAS WI (⭤ )  YPAS MI (⭤ )  YPAS StI (⭤ )  YPAS SI (⭤ )  YPAS TAI (⭤ ) |
| Duncan, M. J., et al., 2011 (UK) [46] | 20 | 20 | 50 | NSC | -- / 2 | -- / 6 | Xbox 360 and Gamercize Power Stepper | Sega Superstars Tennis / FIFA 09 / NSC50 / Proevolution Soccer / Sega Rally / Lego Batman | Conventional | Pedometer (O)  Monitor (O) | Steps/min. (⭤ )  % time MVPA  (⭤ ) |
| Fu, Y., et al., 2018a (USA) [47] | 33 | 32 | 50 | NSC | 30 / 3 | 1620 / 18 | Computer^!^ | GoNoodle / Adventure to Fitness / Cosmic Kids Yoga | Conventional | Pedometer (O) | Step counts (⭡) |
| Fu, Y., et al., 2018b (USA) [48] | 35 | 29 | 52 | NSC | 30 / 5 | 1800 / 12 | Computer^!^ | GoNoodle / Adventure to Fitness / Cosmic Kids Yoga | Nothing | Pedometer (O) | Step counts (⭡) |
| Gao, Z., et al., 2019a (USA) [49] | 18 | 14 | 50 | NSC | 30 / 5 | 1800 / 12 | Leap TV system | LeapTV games | Nothing | AM (O) | Energy expenditure (⭤ ) |
| Gao, Z., et al., 2019b (USA) [50] | 36 | 45 | 52 | NSC | 50 / 1 | 3897 / 39 | Kinect for Xbox 360 and Wii | Just Dance / Wii Fit / Gold's Gym Cardio Workout / Kinect Sports | Nothing | AM (O) | METs (⭡);  Kcal/ day (⭡) |
| Gao, Z., et al. 2014 (USA)* [51] | 72 | 113 | 57 | NSC | 90 / 1 | 3507 / 39 | Playstation 2 DDR System | Dance Dance Revolution | Nothing | Sports, Play, Active Recreation for Kids (SPARK) activity checklist (S) | METs (⭡) |
| Gao, Z., et al., 2019c (USA) [52] | 20 | 36 | 49 | NSC | 20 / 5 | 800 / 8 | Wii and Kinect for Xbox 360 | Wii Just Dance for Kids / Wii Nickelodeon Fit / Xbox Just Dance for Kids | Nothing | AM (O) | MVPA min/day (⭡) |
| Hamari, L., et al., 2019 (Finland)* [53] | 12 | 18 | 72 | Cancer | 30 / 7 | 1680 / 8 | Wii (U) | Hola hoop / Jogging / Island Cycling / Rhythm Kung-Fu | Conventional | AM (O)  Activity diary (S)  MET questionnaire (S) | PA counts/hr (⭤ )  PA min/day (⭤ )  PA MET hr/week (⭤ ) |
| Howie, E. K., et al., 2016 (Australia) [54] | 21 | 21 | 48 | Develop-mental coordination disorder | 20 / 4 | 1280 / 16 | Playstation 3 with Move and Eye Input devices and Kinect for Xbox 360 | Kinect Adventures / Start the Party / Eye Pet / Sports Champions | Nothing | AM (O) | Sedentary min/ day (⭤ )  Light PA min/ day (⭤ )  Moderate PA min/day (⭤ )  Vigorous PA min/day (⭤ ) |
| Imam, B., et al., 2017 (Canada) [55] | 14 | 14 | 64 | Older adults with lower limb amputation | 40 / 3 | 480 / 4 | Wii Fit (Wii fit board and Wii Mote) | Wii Fit | Sedentary (cognitive)  Games | Pedometer (O) | Pedometer (⭤ ) |
| Jung, J., et al., 2009 (Singapore) [56] | 30 | 15 | --- | NSC | 15 / 3 | 270 / 6 | Wii (U) | Wii Sports and Cooking Mama | Sedentary Games | Physical Activity Questionnaire for Elderly Japanese (S) | METs (⭡) |
| Lau, P. W. C., et al., 2016 (China) [57] | 40 | 40 | 69 | NSC | 60 / 2 | 1440 / 12 | Kinect for Xbox 360 | Chose 1 of the 12 offered sports in Season 1 or Season 2 within a play session | Nothing | AM (O) | Average daily total PA (⭡)  Average daily MVPA (⭡) |
| Lwin, M. O., et al., 2012 (Singapore) [58] | 557 | 555 | 54 | NSC | 60 / 1 | 360 / 6 | Wii (U) | Wii tennis and Wii boxing | Conventional | Godin and Shephard’s  leisure time exercise questionnaire (S) | Strenuous exercise (⭡)  Moderate exercise (⭡)  Mild exercise (⭡) |
| Maddison, R., et al., 2011 (New Zealand) [59] | 115 | 116 | 73 | NSC | 8.6 / 7 | -- / 24 | Playstation 2 or 3 EyeToy | EyeToy camera, dance mat, and a selection of active video games (e.g., Play3, Kinetic, Sport, and Dance Factory) | Nothing | AM (O) | Avg daily time (min) spent in  moderate-to-vigorous activities (⭤ ) |
| Maloney, A. E., et al., 2008 (USA) [60] | 40 | 20 | 50 | NSC | -- | -- / 10 | Playstation 2 DDR System | DDR MAX2 game and two padded dance mats | Nothing | AM (O) | Vigorous PA (⭤ )  Light PA (⭤ ) |
| Maloney, A. E., et al., 2012 (USA)** [61] | 33 | 31 | 48 | NSC | -- | -- / 20 | Playstation 2 DDR System | Dance Dance Revolution | Nothing | Godin-Shepard  physical activity questionnaire for children (S)  Pedometer (O)  AM (O) | SR PA (⭤ )  Pedometer steps (⭤ )  AM light, moderate, vigorous (⭤ ) |
| Mhurchu, C.N. et al., 2008 (Canada)* [62] | 10 | 10 | 60 | NSC | -- | -- / 12 | Playstation 2 and EyeToy | EyeToy active games, and dance mat | Conventional | Physical Activity Questionnaire for Children; PAQ-C (S) AM (O) | PAQ-C score (⭤ )  AM counts (⭡)  Time spent light, moderate, vigorous (⭤ ) |
| Ruivo, J. M. A. S., et al., 2017 (Ireland)* [63] | 16 | 16 | 81 | Cardiac rehabilitation | 60 / 2 | 720 / 6 | Wii (U) | Nintendo Wii Sports boxing and canoeing | Conventional | AM (O) | Counts/minute (⭡) |
| Şimşek, T. T., et al., 2016 (Turkey) [64] | 20 | 22 | 69 | Stroke | 60 / 3 | 1080 / 6 | Wii (U) | tennis, punch out, tightrope tension, tilt table and heading | Conventional | Functional  Independence Measure (S) | FIM score (⭤ ) |
| Staiano, A. E., et al., 2017 (USA)* [65] | 19 | 18 | 0 | Overweight/Obese | 60 / 3 | 2160 / 12 | Kinect for Xbox 360 | Just Dance, Dance central | Nothing | AM (O)  Godin-Shephard  Leisure-Time Physical Activity Questionnaire (S) | AM estimated time light, moderate, vigorous PA (⭤ )  SR time light, moderate, vigorous PA (⭤ ) |
| Taylor, L., et al., 2018 (Australia)* [66] | 29 | 36 | 25 | NSC | 35 / 2 | 560 / 8 | Kinect for Xbox 360 | Your Shape Fitness Evolved, Aging with Grace | Conventional | de Morton Mobility Index (S) | DEMMI scores  (⭤ ) |
| Trost, S. G., et al., 2014 (USA) [67] | 34 | 41 | 43 | Overweight/Obese | --- | --- | Kinect for Xbox 360 | Kinect Adventures!; Kinect Sports | Conventional | AM (O) | MVPA (⭡)  VPA (⭡) |
| Ye, S. Y., et al., 2019 (USA) [68] | 36 | 45 | 48 | NSC | 50 / 1 | 1732 / 35 | Kinect for Xbox 360 and Wii | Just Dance, Wii Fit, Gold’s Gym Cardio Workout, and Kinect Sports | Nothing | AM (O) | LPA ($\downarrow$)  MVPA (⭡) |

*Notes*. The sample size corresponds to the final sample used for statistical analysis. The number of men and women corresponds to the enrolled sample, which does not always match the final sample size.

*Legend.*

“---” = missing or not applicable

^!^ = Windows/Mac/Linux/Sun/Other

AM = Accelerometer

MI: Moving index

NSC = No Specific Condition

O = Objective PA measure

S = Subjective PA measure

SI: Sitting Index

StI: Standing Index

SR = Self-reported

TAI: Total Activity Index

TTI = Weekly Total Time Index (h/week)

TEEI = Weekly Total Energy Expenditure Index (MET*h/week)

VI = Vigorous Index

WI = Walking index

⭡ = Significantly better

⭤ = Nonsignificant

$\downarrow$= Significantly worse

** = Contains both Subjective and Objective PA measures and thus were pooled twice in meta-analysis
