# Supplementary material for: Active Video Game Interventions Targeting Physical Activity Behaviors: Systematic Review and Meta-analysis
Source: J Med Internet Res. 2023 May 16;25:e45243. doi: 10.2196/45243 (PMC10230359; doi:10.2196/45243)
Supplement: Multimedia Appendix 4 [file jmir_v25i1e45243_app4.docx]

|  | | GRADE Scores  (-1 =high risk of bias, +1 =low risk of bias) | | | | | | | |
| --- | --- | --- | --- | --- | --- | --- | --- | --- | --- |
| **Study** | Included in meta-analysis | Random assign-ment | Allocation concealment | Blinding of participants and personnel | Blinding of outcome assessment | Complete-ness of reporting outcome data | Selectivity in  reporting | Adjustment for confound-ing variables | Average GRADE score |
| Bock B. C. et al. (2019) [44] | yes | -1 | -1 | 1 | -1 | -1 | -1 | -1 | -.71 |
| Carrasco M. et al. (2020) [45] | yes | 1 | 1 | 0 | 0 | -1 | -1 | -1 | -.14 |
| Duncan M. J. et al. (2011) [46] | yes | -1 | 1 | 1 | 1 | -1 | -1 | -1 | -.14 |
| Fu Y. et al. (2018a) [47] | yes | 1 | 1 | 1 | 0 | -1 | -1 | 1 | .29 |
| Fu Y. et al. (2018b) [48] | yes | -1 | 1 | 1 | 1 | -1 | -1 | 1 | .14 |
| Gao Z. et al. (2019a) [49] | yes | 0 | -1 | 1 | -1 | -1 | -1 | 1 | -.29 |
| Gao Z. et al. (2019b) [50] | yes | 1 | 1 | 1 | 1 | -1 | -1 | 1 | .43 |
| Gao Z. et al. (2014) [51] | no | 1 | 1 | 1 | 1 | -1 | -1 | 1 | .43 |
| Gao Z. et al. (2019c) [52] | yes | 1 | 1 | 1 | 1 | -1 | -1 | -1 | .14 |
| Hamari et al. (2019) [53] | no | -1 | 1 | 1 | -1 | 1 | -1 | 1 | .14 |
| Howie E. K. et al. (2016) [54] | yes | -1 | 0 | 1 | 1 | -1 | -1 | 1 | .00 |
| Imam B. et al. (2017) [55] | yes | -1 | -1 | -1 | -1 | 0 | 0 | -1 | -.71 |
| Jung J. et al. (2009) [56] | yes | -1 | 1 | 1 | 1 | -1 | 0 | 0 | .14 |
| Lau P. W. C. et al. (2016) [57] | yes | -1 | -1 | 0 | 0 | -1 | -1 | 0 | -.57 |
| Lwin M. O. et al. (2012) [58] | yes | -1 | 1 | 1 | 1 | -1 | -1 | 0 | .00 |
| Maddison R. et al. (2011) [59] | yes | -1 | -1 | 1 | 1 | -1 | -1 | 0 | -.29 |
| Maloney A. E. et al. (2008) [60] | yes | -1 | 1 | 0 | 0 | 0 | -1 | 0 | -.14 |
| Maloney A. E. et al. (2012) [61] | yes | -1 | -1 | 1 | 1 | 0 | 0 | -1 | -.14 |
| Mhurchu C. N. et al. (2008) [62] | yes | -1 | 0 | 0 | 0 | -1 | 0 | 0 | -.29 |
| Ruivo J. M. A. S. et al. (2017) [63] | no | -1 | -1 | 1 | -1 | -1 | -1 | -1 | -.71 |
| Şimşek T. T. et al. (2016) [64] | no | -1 | -1 | 1 | -1 | -1 | 0 | -1 | -.57 |
| Staiano A. E. et al. (2017) [65] | yes | -1 | -1 | 1 | -1 | -1 | -1 | -1 | -.71 |
| Taylor L. et al. (2018) [66] | no | -1 | -1 | 1 | -1 | -1 | -1 | -1 | -.71 |
| Trost S. G. et al. (2014) [67] | no | -1 | 1 | 1 | 1 | -1 | -1 | -1 | -.14 |
| Ye, S. Y. et al. (2019) [68] | yes | -1 | 0 | 0 | 0 | -1 | 0 | -1 | -.43 |

GRADE = Grading of Recommendations Assessment, Development and Evaluation (GRADE) assessment. The six components evaluated include random assignment (to avoid selection bias), allocation concealment (to avoid selection bias), blinding of participants and personnel (to avoid performance bias), blinding of outcome assessment (to avoid detection bias), completeness of reporting of some outcome data (to avoid attrition bias), and selectivity in reporting (to avoid reporting bias). We added a seventh criterion, which assessed any potential adjustment for additional confounding variables. Each category was scored from -1 to +1, where -1 represented high risk of bias, 0 represented unclear risk, and 1 represented low risk of bias.
